# Combining an Evolution-guided Clustering Algorithm and Haplotype-based LRT in Family Association Studies

**DOI:** 10.1186/1471-2156-12-48

**Published:** 2011-05-19

**Authors:** Mei-Hsien Lee, Jung-Ying Tzeng, Su-Yun Huang, Chuhsing Kate Hsiao

**Affiliations:** 1 Department of Mathematics and Computer Science Education, Taipei Municipal University of Education, Taipei 10048, Taiwan; 2 Department of Statistics and Bioinformatics Research Center, North Carolina State University, Raleigh, North Carolina 27695, USA; 3 Institute of Statistical Science, Academia Sinica, Taipei 11529, Taiwan; 4 Department of Public Health and Institute of Epidemiology and Preventive Medicine, National Taiwan University, Taipei 10055, Taiwan; 5 Bioinformatics and Biostatistics Core, NTU Center for Genomic Medicine, National Taiwan University, Taipei 10055, Taiwan; 6 Research Center for Gene, Environment, and Human Health, College of Public Health, National Taiwan University, Taipei 10055, Taiwan

## Abstract

**Background:**

With the completion of the international HapMap project, many studies have been conducted to investigate the association between complex diseases and haplotype variants. Such haplotype-based association studies, however, often face two difficulties; one is the large number of haplotype configurations in the chromosome region under study, and the other is the ambiguity in haplotype phase when only genotype data are observed. The latter complexity may be handled based on an EM algorithm with family data incorporated, whereas the former can be more problematic, especially when haplotypes of rare frequencies are involved. Here based on family data we propose to cluster long haplotypes of linked SNPs in a biological sense, so that the number of haplotypes can be reduced and the power of statistical tests of association can be increased.

**Results:**

In this paper we employ family genotype data and combine a clustering scheme with a likelihood ratio statistic to test the association between quantitative phenotypes and haplotype variants. Haplotypes are first grouped based on their evolutionary closeness to establish a set containing core haplotypes. Then, we construct for each family the transmission and non-transmission phase in terms of these core haplotypes, taking into account simultaneously the phase ambiguity as weights. The likelihood ratio test (LRT) is next conducted with these weighted and clustered haplotypes to test for association with disease. This combination of evolution-guided haplotype clustering and weighted assignment in LRT is able, via its core-coding system, to incorporate into analysis both haplotype phase ambiguity and transmission uncertainty. Simulation studies show that this proposed procedure is more informative and powerful than three family-based association tests, FAMHAP, FBAT, and an LRT with a group consisting exclusively of rare haplotypes.

**Conclusions:**

The proposed procedure takes into account the uncertainty in phase determination and in transmission, utilizes the evolutionary information contained in haplotypes, reduces the dimension in haplotype space and the degrees of freedom in tests, and performs better in association studies. This evolution-guided clustering procedure is particularly useful for long haplotypes containing linked SNPs, and is applicable to other haplotype-based association tests. This procedure is now implemented in R and is free for download.

## Background

High-density sets of SNPs, especially haplotypes, have been used widely in genetic research to explore possible association with complex diseases. Haplotypes are considered to be the biological units containing more information about transmission, and thus may be better biomarkers to use in examining the disease susceptible region. However, haplotype phase is often unknown when only genotype data are observed. This linkage phase ambiguity often leads to large degrees of freedom in statistical tests, and may result in estimation of many haplotypes with rare frequencies. Collection of family genotype data may help in determination of haplotype phase if information from other family members can be incorporated and cross-referenced. Additionally, the use of family data can avoid spurious association arising from population admixture. Nevertheless, the statistical analysis of family data may not be straightforward. For instance, the nonparametric transmission disequilibrium test (TDT) and other similar tests utilize the transmitted and non-transmitted alleles (or haplotypes) to detect association. For uncertainty both in transmission and in phase, most procedures adopt an expectation-maximization (EM) algorithm in computation of score statistics or likelihood functions, such as FBAT [1] and FAMHAP [2,3]. These methods infer haplotypes based on nuclear families, where any number of children and configuration of missing genotype data are allowed. FBAT considers score statistics for the number of risk haplotypes among affected offspring; whereas FAMHAP adopts a likelihood approach to estimate haplotype frequencies and to test for association based on nuclear family members or unrelated individuals.

As for addressing the problems that result when a large number of different haplotypes are involved in analysis and when certain haplotypes occur with very low frequency, several approaches have been adopted. Some studies have deleted haplotypes with small estimated frequencies [4,5], and some have combined these rare haplotypes to form a new group. Although these procedures can reduce the total number of parameters and the inflated variation due to small frequencies, they risk information loss due to the arbitrary deletion or grouping of rare haplotypes. In contrast, several procedures have adopted an approach which uses a measure of evolutionary relationship to cluster rare haplotypes in case-control studies [6-10]. These clustering algorithms define a core set of haplotypes that are considered "ancient," where being ancient is approximated by being more frequent [7-10]. Once the core set is determined, rare haplotypes are then clustered with their ancestors as well as denoted by them. For family data, we want to employ such a clustering scheme, so that the statistical tests can be conducted more efficiently.

Under the assumption of random mating and with the use of transmitted and non-transmitted haplotypes from parents, we construct for family data a core set of haplotypes based on estimates of haplotype frequencies. In the following sections, we start with the notation used in Becker and Knapp [2], conduct the clustering procedure for family data based on frequency estimates from FAMHAP, determine the pair of transmitted and non-transmitted core-coding haplotypes, and introduce the assignment of coding to be used later in our tests of disease association. The uncertainty in phase explanation, in transmission status, and in core representation associated with each genotype will be expressed with weights. Unlike the analysis for case-control studies, these weights are essential for further statistical analysis of pedigree data. We next adopt this transformed data in a likelihood ratio test (LRT) of association, and call it LRT-C. This LRT-C modifies the original test in FAMHAP in terms of the core haplotypes. To evaluate if the proposed clustering approach can identify correctly the true core haplotypes and to demonstrate the advantage of LRT-C accrued by inclusion of an evolutionary interpretation, we conduct simulation studies by applying a coalescent-based whole genome simulator GENOME [11]. For the purpose of comparison with other tests, we consider the original LRT in FAMHAP, a score test in FBAT, and a naïve LRT with all rare haplotypes clustered to form a new group (LRT-G).

## Methods

### Notation

Following the same notation used in Becker and Knapp [2], let *G_i _*denote the set of unphased genotypes in the *i*-th family, , where the superscripts index the members in the *i*-th family. For example,  and  are multilocus genotypes of father and mother, respectively, and  denotes the genotype of the *l*-th child, where 1 ≤*l*≤*n_i _*and *n_i _*is the number of children in the *i*-th family. If haplotypes *j *and *k *are compatible with the father's genotype , this relation is denoted by . Similarly, if haplotypes *u *and *v *are compatible with the mother's genotype , it is then denoted by . The rest are deduced analogously. Furthermore, if the *l*-th child is the proband in the *i*-th family, then his/her haplotype transmission patterns will be inferred with reference to each sibling's genotype information as well as the parents' information. Therefore, we define  as the haplotype explanation set containing all possible haplotype transmission patterns compatible with, *G_i_*,

where  represents the number of transmission patterns of (*j*, *k*, *u*, *v*) that are compatible with the genotype  of the child *l'*.

It is worth noting that, under the null hypothesis of no association,  is the intersection of all  for *l' *= 1,...,*n_i _*in the *i*-th family. Consequently, the estimation of haplotype frequency is independent of who the proband is. In contrast, when the gene is associated with the disease, the haplotype explanation set  and the estimations of the transmitted and non-transmitted haplotype frequencies will depend on the ascertained proband. Therefore, throughout this paper, we rearrange the order of children such that the first child is always the first affected one in each family. In the following, we use FAMHAP to estimate the configurations of haplotypes and their corresponding frequencies based on likelihoods. In fact, at this stage of computation, other public software programs like FBAT and Transmit [12] can provide stable estimates as well.

### Step 1 Clustering haplotypes

With the frequency approximating the "age" of haplotypes, a cladistic clustering approach is conducted based on their evolutionary relation. Similar to the haplotype clustering method for case-control studies in Tzeng [6], here we identify first the ancestor (core) and descendent haplotypes based on family data, and then cluster the descendents with their ancestors for dimension reduction.

The selected core haplotypes are the leading *c *haplotypes with a cumulative sum of frequencies reaching 90%, and are defined as the set  with corresponding frequencies

The superscript is the number of generations in the evolutionary tree, and the subscript stands for the order of haplotype frequency from large to small. Similarly, the set of haplotypes with one step mutation from *H*^(0) ^is denoted as *H*^(1)^, and those *m *steps away are contained in *H*^(*m*)^. For each set *H*^(*m*)^, the corresponding haplotype frequencies are denoted as ∏^(*m*)^, where *m *= 1,...,*M *and *M *is the largest distance. Between any two adjacent sets *H*^(*m*) ^and *H*^(*m*-1)^, an allocation matrix **B**^(*m*) ^is defined to represent the probability that a certain haplotype in *H*^(*m*) ^is a direct descendant of a haplotype in *H*^(*m*-1) ^[6,13]. Therefore, the frequencies of the core haplotypes can be revised as

where (∏^(*m*)^)^*t *^is the transpose of ∏^(*m*)^. Detailed explanation is provided in Additional file 1.

Note that the above derivations do not require the information of haplotype phase for each family member. In other words, any software which provides estimates of haplotype frequencies can be applied at this stage. However, we prefer FAMHAP because it also computes for each family the compatible transmitted and non-transmitted haplotypes, along with the weights, which will be utilized in the following steps of recoding and testing.

### Step 2 Recoding transmission and non-transmission haplotypes for analysis

After determining the core haplotypes with updated frequencies, we begin to *rewrite *the remaining non-core haplotypes in terms of their corresponding ancestor haplotypes in the core. In other words, all representations including haplotype explanation, phase uncertainty, and transmission stages are rewritten as a function of the core in order to reduce dimensionality. This recoding procedure can be carried out via the allocation matrix **B**^(*m*) ^defined earlier. Let matrix Γ^*m *^be the product of *m *matrices **B**^(*m*)^,**B**^(*m*-1)^,... and **B**^(1)^, thus the row dimension of Γ^*m *^indicates the number of rare haplotypes in *H*^(*m*)^, and the column dimension of Γ^*m *^stands for the number of core haplotypes in *H*^(0)^. For instance, the *i*-th row in Γ^*m *^is(1)

where *c *this -dimensional row vector lists the probabilities that the *i*-th haplotype in *H*^(*m*) ^is to be clustered with the ancestor haplotypes in the core. Therefore, in the following the original frequency  of a rare haplotype is replaced by , a linearly weighted sum of the modified core haplotype frequencies(2)

The transmission status, along with its probability, haplotype phase ambiguity, and evolution uncertainty, can now be re-arranged, before statistical analysis, using equation (2). For instance, under the alternative hypothesis of association, the frequency of the *i*-th haplotype in *H*^(*m*) ^for the transmission group *Tr *becomes , where  is the updated core haplotype frequency vector

and  is the frequency vector of transmitted haplotypes in *H*^(*m*)^. For the non-transmission group *NTr*, the calculations of  and  are carried out in the same way.

### Step 3 Likelihood ratio test with clustered haplotypes (LRT-C)

We now derive the likelihood ratio test statistic with all parameters rewritten in terms of the core haplotype frequencies. This amounts to rewriting Becker and Knapp's [14] procedure with only (*c *- 1) parameters,

where *n *is the number of families and  is the likelihood of the *i*-th family under the alternative with the first child being affected

The *γ_j.Tr _*and *γ_u.Tr _*stand for frequencies in the new representation (2) for the transmitted haplotypes (*j,u*), and *γ_k.NTr _*and *γ_v.NTr _*are the modified frequencies for the non-transmitted haplotypes (*k,v*). The function *s *is the number of transmission patterns compatible with the genotype  of the *l'*-th child *c_l'_*, as defined in Becker and Knapp's paper. Note that the value of will not differ no matter what haplotypes, core or not, are being investigated. That is, there is no need to recompute *s *with respect to the core system, and its value derived when specifying the haplotype explanation set  in the beginning of this procedure can be used directly here.

## Results

To evaluate the performance of the likelihood ratio test with clustered haplotypes in family studies, we conduct simulations to first examine the reconstruction and identification of core haplotypes, and next to evaluate the impact of this clustering scheme on the likelihood ratio test. The results are compared with three family-based association methods, FAMHAP [2], FBAT [15], and finally an LRT using a naïve group composed exclusively of rare haplotypes (LRT-G).

### Sampling scheme for simulations

**Figure 1:**
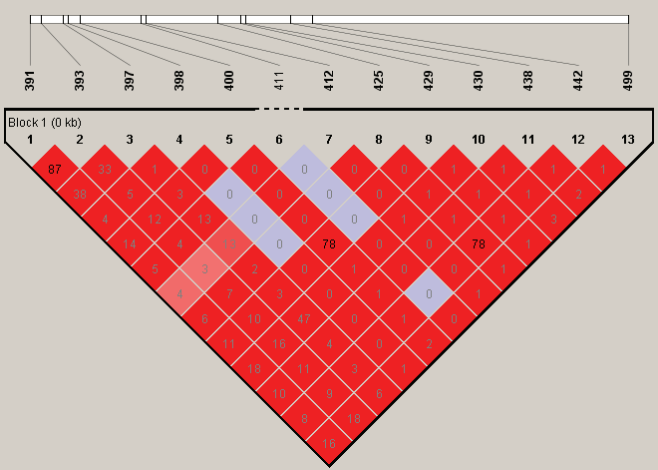
**LD plot**. LD plot of the simulated region.

**Figure 2:**
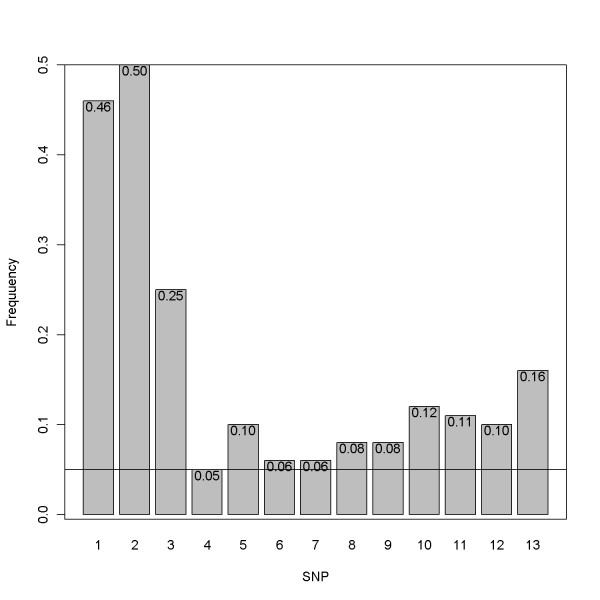
**Minor allele frequencies**. Minor allele frequencies of the 13 SNPs consisting of the haplotype region.

**Figure 3:**
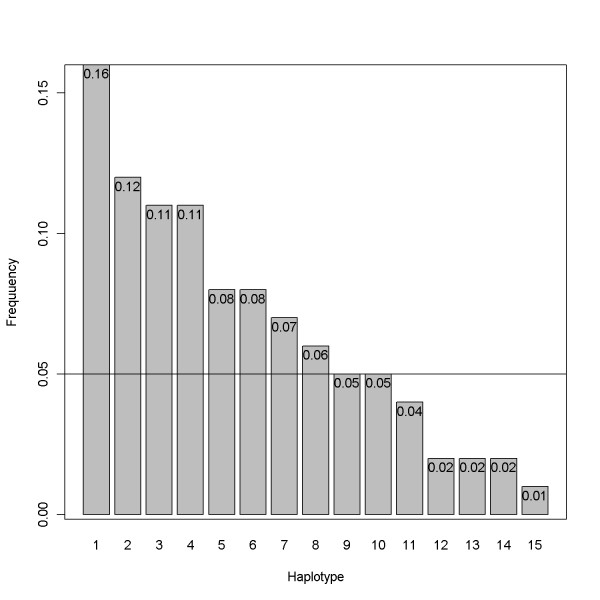
**Haplotype frequencies**. Frequencies of the 15 haplotypes considered in simulation studies.

The SNP haplotype sequences were first simulated based on a coalescent-based whole genome simulator GENOME [11]. These sequences were generated from a population with an effective size of 10,000, with the number of SNPs assumed to follow a Poisson distribution with mean equal to the product of mutation rate 10^-6^/bp, and with 1,000 base pairs per each fragment. The recombination rate between ten consecutive fragments was assumed to be 10^-4^. Default settings were used for other parameters such as the mutation and migration rates of 10^-6 ^and 2.5 × 10^-4^, respectively. This resulted in 100 sequences with 972 SNPs. After deleting alleles with minor allele frequency (MAF) less than 5%, 536 SNPs were left. Haploview [16] was next used to identify haplotype blocks and to extract tag SNPs. Finally, seven blocks were determined. We selected the longest block, the seventh, for use in constructing the data for nuclear families and derived 13 tag SNPs which formed 15 haplotypes in this region. Figure 1 shows the linkage disequilibrium (LD) plot with haploview. A complete plot of LD for all tag SNPs with corresponding haplotype blocks is displayed in Additional file 2. Figures 2 and 3 display the corresponding minor allele frequencies and the haplotype frequencies, respectively.

In the following simulations the number of families *N *= 200 was considered. For each family, the number of children, in addition to the proband, follows a Poisson distribution with mean 2, and this value was kept the same for this family in each replication. Let one of the marker loci ' *A *' denote the liability allele with frequency *p *fixed at 0.1, 0.25, or 0.5. Let *f_i _*be the penetrance function, where *i *= 0, 1, 2, and let *r *= *f_1_*/*f_0 _*= 2, 2.5, or 3 be the relative ratio. The prevalence *K *was set at 1% under the recessive, additive, and dominant models. These values for simulation settings are listed in Table 1. There were 27 simulation settings considered, and there were 1,000 replications under each setting. For each family, we generated first the haplotypes of the proband, next the parents' non-transmitted haplotypes, and then the haplotypes of other siblings. All haplotype data were transformed to genotypes before analysis.

**Table 1:** Penetrance values for simulation settings

	additive model			dominant model			recessive model		
	**10**^**4 **^**× *f***_**0**_	**10**^**4 **^**× *f***_**1**_	**10**^**4 **^**× *f***_**2**_	**10**^**4 **^**× *f***_**0**_	**10**^**4 **^**× *f***_**1**_	**10**^**4 **^**× *f***_**2**_	**10**^**4 **^**× *f***_**0**_	**10**^**4 **^**× *f***_**1**_	**10**^**4 **^**× *f***_**2**_

*r *= 3									
*p *= 0.1	71	214	357	72	217	217	98	98	294
*p *= 0.25	50	150	250	53	160	160	89	89	267
*p *= 0.5	33	100	167	40	120	120	67	67	200
									
*r *= 2.5									
*p *= 0.1	77	192	308	78	195	195	99	99	246
*p *= 0.25	57	143	229	60	151	151	91	91	229
*p *= 0.5	40	100	160	47	118	118	73	73	182
									
*r *= 2									
*p *= 0.1	83	167	250	84	168	168	99	99	198
*p *= 0.25	67	133	200	70	139	139	94	94	188
*p *= 0.5	50	100	150	57	114	114	80	80	160

Numbers listed are scaled penetrance values (10^4 ^× *f*_0_, 10^4 ^× *f*_1_, 10^4 ^× *f*_2_) corresponding to different allele frequencies (*p*) and relative ratios (*r*) under three genetic models

The prevalence *K *is set at 0.01

### Identification of core haplotypes and tests of association

**Figure 4:**
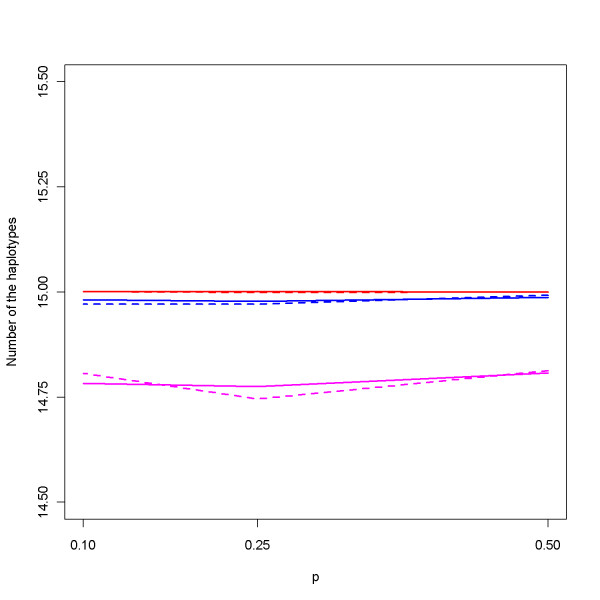
**Average number of identified haplotypes under the additive model**. The number of haplotypes identified in the simulations under various numbers of families (*N*), relative ratios (*r*), and liability allele frequencies (*p*) under additive models. Red lines are for *N *= 200 families, blue for *N *= 100 families, and pink for *N *= 50. Solid lines are for *r *= 2 and dashed lines for *r *= 3.

**Figure 5:**
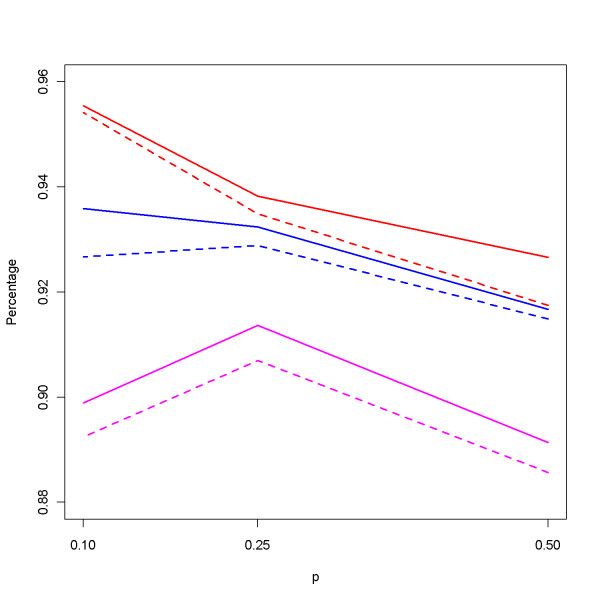
**Average of percentage of identified core haplotypes**. The average percentage of identified core haplotypes among the 10 true core haplotypes under different allele frequencies *p*. Red lines are for *N *= 200 families, blue for *N *= 100 families, and pink for *N *= 50. Solid lines are for *r *= 2 and dashed lines for *r *= 3.

Once the family genotype data were simulated, they were used to estimate haplotype compositions, corresponding frequencies, and also the set of core haplotypes. Figure 4 displays the average number of haplotypes identified in the simulation studies under the additive model for various numbers of families (*N *= 50, 100, and 200) and relative ratios (*r *= 2 and 3). These numbers are all close to the true value 15. Results under the dominant and recessive models are similar (data not shown here). In Figure 5, we plot the average percentage of identified core haplotypes among the set of ten true core haplotypes. The percentages are high indicating good representation and consistency of this evolution-guided clustering procedure for family data. It can be observed in the figure that the percentage of core haplotypes being identified by our procedure is not much affected by the frequency *p *of the susceptible allele *A*, or by the mode of inheritance. For example, under recessive models with *r *= 2 (the solid pink line in Figure 5), the percentages are 90%, 91%, and 89% for *p *= 0.1, 0.25, and 0.50, respectively, corresponding to the average numbers 9.4, 9.4, and 9.2 core haplotypes being identified among the 10 true cores. In addition, when *p *is fixed at 0.5, the percentages are 89%, 90%, and 89% for the additive, dominant, recessive model, respectively, implying robust performance of the evolutionary clustering procedure.

After the core haplotypes are determined, the next step is to construct the likelihoods under the null and the alternative hypotheses, respectively, with all haplotype frequencies replaced by the revised core frequencies. This new modified likelihood ratio test with clustered haplotypes, LRT-C, is compared with a nonparametric score test in FBAT, an LRT with all rare haplotypes grouped into a single class (LRT-G), and an original test in FAMHAP. The resulting powers for *N *= 200 are displayed in Table 2. Under each setting, the largest power among the three tests is indicated in boldface. In most cases, LRT-C is more powerful than the other three tests. In addition, the power gain from LRT-C is substantially pronounced when the relative ratio *r *is large. The power of LRT-G is between that of LRT-C and the original FAMHAP most of the time, whereas FBAT is always the least powerful, except when the allele frequency *p *is 0.1 under the recessive model. Type I errors are presented in Table 3, under the null hypothesis that none of the SNPs (with allele frequency ranging from 0.05 to 0.5) nor the haplotypes (with frequency ranging from 0.01 to 0.16) is associated with the disease. Note that the type I errors of LRT-C, LRT-G and FAMHAP are all around nominal values; while that of FBAT is the smallest.

**Table 2:** Number are the power of four family-based association tests at 5% significance level with *N *= 200

	**Additive model**				**Dominant model**				**Recessive model**			
			
	**LRT-C**	**LRT-G**	**FAMHAP**	**FBAT**	**LRT-C**	**LRT-G**	**FAMHAP**	**FBAT**	**LRT-C**	**LRT-G**	**FAMHAP**	**FBAT**
			
*r *= 3												
*p *= 0.1	**0.956**	0.914	0.931	0.916	**0.898**	0.835	0.860	0.864	0.058	0.059	0.062	**0.087**
*p *= 0.25	**0.997**	0.991	0.990	0.987	**0.926**	0.888	0.863	0.888	**0.382**	0.334	0.341	0.156
*p *= 0.5	**0.944**	0.900	0.896	0.887	**0.409**	0.382	0.353	0.338	**0.941**	0.920	0.909	0.117
												
*r *= 2.5												
*p *= 0.1	**0.815**	0.734	0.786	0.743	**0.737**	0.643	0.683	0.652	0.067	0.063	0.071	**0.083**
*p *= 0.25	**0.952**	0.917	0.912	0.891	**0.804**	0.748	0.730	0.753	**0.256**	0.209	0.212	0.089
*p *= 0.5	**0.852**	0.794	0.798	0.773	**0.322**	0.295	0.277	0.267	**0.805**	0.753	0.726	0.094
												
*r *= 2												
*p *= 0.1	**0.535**	0.438	0.473	0.422	**0.446**	0.381	0.400	0.368	0.049	0.052	**0.054**	**0.054**
*p *= 0.25	**0.769**	0.696	0.676	0.645	**0.519**	0.439	0.452	0.426	**0.153**	0.138	0.141	0.060
*p *= 0.5	**0.698**	0.646	0.607	0.575	**0.201**	0.193	0.192	0.165	**0.490**	0.438	0.444	0.068

**Table 3:** Type I errors of the four family-based association tests at the 5% significance level

	LRT-C	LRT-G	FAMHAP	FBAT
*N *= 200	0.044	0.045	0.051	0.038

**Figure 6:**
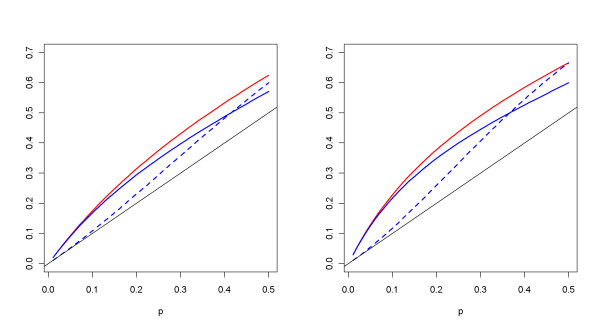
**Plots of *P*(*A*|*D*) versus *p *under three genetic models**. The values of *P*(*A*|*D*) correspond to different *p *'s under additive model (red line), dominant model (solid blue line), and recessive model (dashed blue line). The left panel is for *r *= 2 **(Left) **and the right for *r *= 3 **(Right)**. The straight black line indicates when magnitudes of *P*(*A*|*D*) and *p *are equal.

The above Table 2 seems to indicate a poor relationship between the power performance and the allele frequency *p *= *P*(*A*) under the recessive model. For instance, when *p *= 0.1, the power under the additive and dominant model is satisfactory; while the power under the recessive model is only around 0.05. One possible explanation could be the influence from penetrance, prevalence, and *p *on *P*(*A*|*D*), where *P*(*A*|*D*) is the allele frequency among diseased individuals. Because *P*(*A*|*D*) can be written as [*f*_2 _. *p*^2 ^+ *f*_1 _. *p *. (1 - *p*)]/*K *(see Additional file 3 for details), its value is closer to *P*(*A*) under the recessive model. For example, *p*(*A*) = 0.1 corresponds to *P*(*A*|*D*) = 0.11 when *r *= 2, which indicates that the allele *A *and disease status *D *are close to independent. In other words, it is unlikely in this case that the allele *A *is the susceptible marker and, therefore, the power becomes low. In contrast, under the additive model, *P*(*A*|*D*) is 0.21 if *r *= 2 and *p *= 0.12. In this case, the allele *A *may be the susceptible gene and thus the power is larger. More details on the derivation of *P*(*A*|*D*) can be seen in Additional file 3 and the comparison between *P*(*A*|*D*) and *P*(*A*) is displayed in Figure 6. The black solid line in Figure 6 represents the case where the magnitudes of *P*(*A*|*D*) and *P*(*A*) are equal.

### Performance evaluation under population admixture

Another issue of note concerns the effect of population stratification on the power of LRT-C. To investigate this effect, we performed further simulation studies with data generated from two populations via GENOME. Similar to the procedures described above, we extracted tag SNPs with Haploview and selected a block with 13 tagSNPs to construct genotype data for *N *= 200 families with 100 families from each population. The liability allele frequency *p *was set at 0.085, 0.270 and 0.530, corresponding to allele frequencies (0.09, 0.43, 0.51) for the first population and (0.08, 0.11, 0.55) for the second. The number of children, penetrance function, relative ratio, genetic models and prevalence were the same as previously defined. Table 4 lists the power of the four association tests under the population admixture data, where LRT-C is the most powerful and FBAT has the smallest power. This result demonstrates that the effect of population admixture on LRT-C is much smaller than it is on either FAMHAP or TRANSMIT, whereas FBAT and the HAP-TDT option in FAMHAP [14,17] can escape only partially from this admixture problem [18], especially when the mode of inheritance is not additive. In addition, the newly developed feature HAP-TDT in FAMHAP does not allow missing data, which makes it less appealing than the original feature in FAMHAP where missing genotype data is allowed for family members.

**Table 4:** Numbers are the power of four family-based association tests for population stratification data at the 5% significance level with *N *= 200

	**Additive model**				**Dominant model**				**Recessive model**			
			
	**LRT-C**	**LRT-G**	**FAMHAP**	**FBAT**	**LRT-C**	**LRT-G**	**FAMHAP**	**FBAT**	**LRT-C**	**LRT-G**	**FAMHAP**	**FAT**
			
*r *= 3												
= 0.085 (0.09, 0.08)	**0.918**	0.903	0.868	0.793	**0.860**	0.855	0.771	0.716	0.057	0.066	**0.091**	0.061
= 0.27 (0.43, 0.11)	**0.908**	0.850	0.880	0.838	**0.644**	0.573	0.638	0.580	**0.348**	0.309	0.343	0.036
= 0.53 (0.51, 0.55)	**0.847**	0.838	0.819	0.710	0.273	**0.279**	0.268	0.188	0.877	**0.884**	0.857	0.065
												
*r *= 2.5												
= 0.085 (0.09, 0.08)	**0.739**	0.727	0.678	0.519	**0.651**	0.640	0.564	0.464	0.046	0.055	**0.076**	0.054
= 0.27 (0.43, 0.11)	**0.778**	0.702	0.752	0.682	0.442	0.380	**0.470**	0.352	0.217	0.198	**0.232**	0.046
= 0.53 (0.51, 0.55)	0.747	**0.748**	0.728	0.595	0.195	0.208	**0.224**	0.133	**0.724**	0.705	0.686	0.069
												
*r *= 2												
= 0.085 (0.09, 0.08)	**0.446**	**0.446**	0.400	0.261	**0.361**	0.346	0.337	0.233	0.040	**0.044**	0.078	0.046
= 0.27 (0.43, 0.11)	0.448	0.404	**0.459**	0.354	0.234	0.210	**0.267**	0.171	**0.109**	0.101	0.143	0.028
= 0.53 (0.51, 0.55)	**0.544**	0.527	0.507	0.355	**0.142**	0.137	0.141	0.091	**0.370**	0.362	0.359	0.041

The two proportions in parentheses (in the first column) indicate the two frequencies under each population, and  is their average

## Discussion

In this paper, we have constructed a family-based association test using clustered haplotypes. The four key steps are: (1) to determine the core set on the basis of haplotype frequencies, (2) to perform the clustering procedure based on a haplotype cladogram, (3) to represent the rare haplotype frequencies in terms of the revised core frequencies, and (4) to incorporate the phase ambiguity, transmission uncertainty, and core-representation variability via likelihood weights. Our simulations show that both haplotype reconstruction and core identification perform well with more than 91% accuracy for cases where number of families *N *≥ 100. In addition, the results show great improvement in test power, as compared to the original FAMHAP, FBAT, and LRT-G. Our proposed procedure is useful for long haplotypes containing many SNPs in LD. If the SNPs are not in LD, then it is not appropriate to consider haplotypes as the unit for analysis. In addition, if the haplotypes are of short length, then both the dimensionality and phase ambiguity will not be hard to handle. We have also embedded this clustering algorithm in the likelihood ratio test under FAMHAP, and this program can be downloaded freely from the author's website http://homepage.ntu.edu.tw/~ckhsiao/download(en).html.

One issue that merits discussion concerns the number of haplotypes in the core set. As a rule of thumb, we selected the several leading haplotypes with 0.9 cumulative frequency. This choice is somewhat arbitrary. In fact, there is a trade-off between the increase in information (represented via frequency) and the reduction in dimensionality. A possible alternative, depending on the sample size and number of dimensions under consideration, would be to use Shannon's information with a penalty function. This criterion works by finding *l *haplotypes such that Shannon's net information reaches its maximum. This criterion, however, is sensitive with respect to sample size. When sample size gets large, the penalty decreases faster than the entropy term, and thus results in inclusion of all haplotypes in the core set, even those with small frequencies. In other words, this criterion does not effectively reduce dimensionality when large sample size prevails.

There are several potential applications for the association test presented here. In many association tests, the chi-square approximation can be poor due to the existence of many haplotypes and/or rare haplotypes. Our clustering procedure may improve the performance of such statistical methods. Although we only demonstrate its impact on the likelihood ratio test, we believe other tests would benefit from this clustering procedure as well. For instance, after the haplotype phase and transmission status are identified and recoded via the core for each family, the tests in TRANSMIT or FBAT or other kinds of haplotype inference [19-22] can be modified. Another application is to use the clustered haplotypes in regression analysis to incorporate the environmental influence on quantitative traits [23,24]. Because FAMHAP provides for each family and its individual family members the list of all possible haplotype explanations with corresponding likelihoods, this information can be utilized in further analyses. In addition, it would be interesting to extend this clustering approach to data containing both independent and related individuals. A mixture of family-based and population-based data are sometimes considered in meta-genetic analysis to enhance the power of an association study. Applying this clustering technique can further reduce the dimension of parameters and achieve a larger power in detection of genetic association with common diseases. Finally, we would like to point out that we consider in this paper only simulations from one single population or from a mixture of two populations. Though the results look promising, other scenarios are warrant for further investigation.

## Conclusions

For family genotype data, we consider an evolution-guided clustering tool that clusters rare haplotypes in order to achieve dimensional reduction, and a parametric likelihood ratio test that accounts for the uncertainty associated with transmission phase. This procedure is able to preserve biological information and to improve statistical testing power. Simulation studies of long haplotypes with SNPs in LD show that the proposed likelihood ratio test with clustered haplotypes (LRT-C) outperforms FAMHAP, FBAT, and a naïve LRT-G.

## Authors' contributions

MHL implemented the algorithm, performed the simulations, and drafted the manuscript. JYT suggested the evolution idea in family-based association study and revised the manuscript. SYH interpreted the concept of likelihood ratio tests and revised the manuscript. CKH conceived the research, supervised the study and finalized the manuscript. All authors read and approved the final manuscript.

## Supplementary Material

Additional file 1 **Derivation of haplotype frequency estimates and haplotype explanation set for each family based on genotype data**. Click here for file

Additional file 2 **The complete plot of LD for all tag SNPs**. Click here for file

Additional file 3 **Derivation of *P*(*A*|*D*)**. Click here for file
