# Supplementary material for: Combining an Evolution-guided Clustering Algorithm and Haplotype-based LRT in Family Association Studies
Source: BMC Genet. 2011 May 19;12:48. doi: 10.1186/1471-2156-12-48 (PMC3118131; doi:10.1186/1471-2156-12-48)
Supplement: Additional file 1 — Derivation of haplotype frequency estimates and haplotype explanation set for each family based on genotype data. [file 1471-2156-12-48-S1.PDF]

Additional file 1

Derivations of haplotype frequency estimates and haplotype explanation set for each family based on genotype data

For family studies, the output of FAMHAP provides two pieces of useful information that can be utilized in our proposed test. One is the haplotype frequency estimate and the other is the set of all possible combinations of transmitted and non-transmitted haplotypes for each family genotype. The following four steps explain our implementation procedures:

Our first step is to sort these haplotype frequency estimates and select the first few, say c, common ones such that their cumulative sum of frequency is not less than 90%. These are called core haplotypes, and form the core set $H^{(0)} = \{h^0_{(1)}, h^0_{(2)}, ..., h^0_{(c)}\}$ with corresponding haplotype frequencies $\prod^{(0)} = \{\pi^0_{(1)}, \pi^0_{(2)}, ..., \pi^0_{(c)}\}$. The superscript is the number of generations in the evolutionary tree, and the subscript stands for the order of haplotypes with frequencies from large to small.

Second, we calculate the "distance" between the core and each of the rest rare haplotypes by counting the number of mutations between them. The set of rare haplotypes with one step mutation from $H^{(0)}$ is denoted as $H^{(1)}$. Similarly, those of two steps mutations are contained in $H^{(2)}$. For each set $H^{(m)}$, the corresponding haplotype frequencies are denoted as $\prod^{(m)}$, where $m = 1, ..., M$, and M is the largest distance.

The third step is to, for any two consecutive generations, establish an allocation matrix $\mathbf{B}^{(m)}$ between $H^{(m)}$ and $H^{(m-1)}$, where each element in $\mathbf{B}^{(m)}$ represents the probability that a certain haplotype in $H^{(m)}$ is a direct descendant of a haplotype in

$H^{(m-1)}$. If a haplotype has more than one direct ancestor, the probabilities will be weighted by the corresponding haplotype frequencies of these ancestor haplotypes in $H^{(m-1)}$. However, if a certain haplotype in $H^{(m)}$ cannot find a one step parent, a pseudo parent will be generated in $H^{(m-1)}$.

Finally, for each descendant, there is a path (or weighted multiple paths) leading to at least one core. This core is where the rare haplotype will be clustered to. In addition, the frequencies of the core haplotypes are revised as

$$(\prod_{core}^{*})^{t} = (\prod^{(0)})^{t} + (\prod^{(1)})^{t}\mathbf{B}^{(1)} + (\prod^{(2)})^{t}\mathbf{B}^{(2)}\mathbf{B}^{(1)} + \cdots + (\prod^{(M)})^{t}\mathbf{B}^{(M)}\mathbf{B}^{(M-1)}\cdots\mathbf{B}^{(2)}\mathbf{B}^{(1)}$$

where $(\prod^{(m)})^{t}$ is the transpose of $\prod^{(m)}$. With the estimate haplotype frequencies, our procedures list the core haplotypes and compute the revised core frequencies.

Although many software provide estimates of haplotype frequencies, here we adopt FAMHAP and add to it these clustering procedures so that, for each family, the transmitted and non-transmitted haplotypes, along with the weights, can be tabulated and further utilized in the recoding steps.
