# Supplementary figures and images for: Combining an Evolution-guided Clustering Algorithm and Haplotype-based LRT in Family Association Studies
Source: BMC Genet. 2011 May 19;12:48. doi: 10.1186/1471-2156-12-48 (PMC3118131; doi:10.1186/1471-2156-12-48)

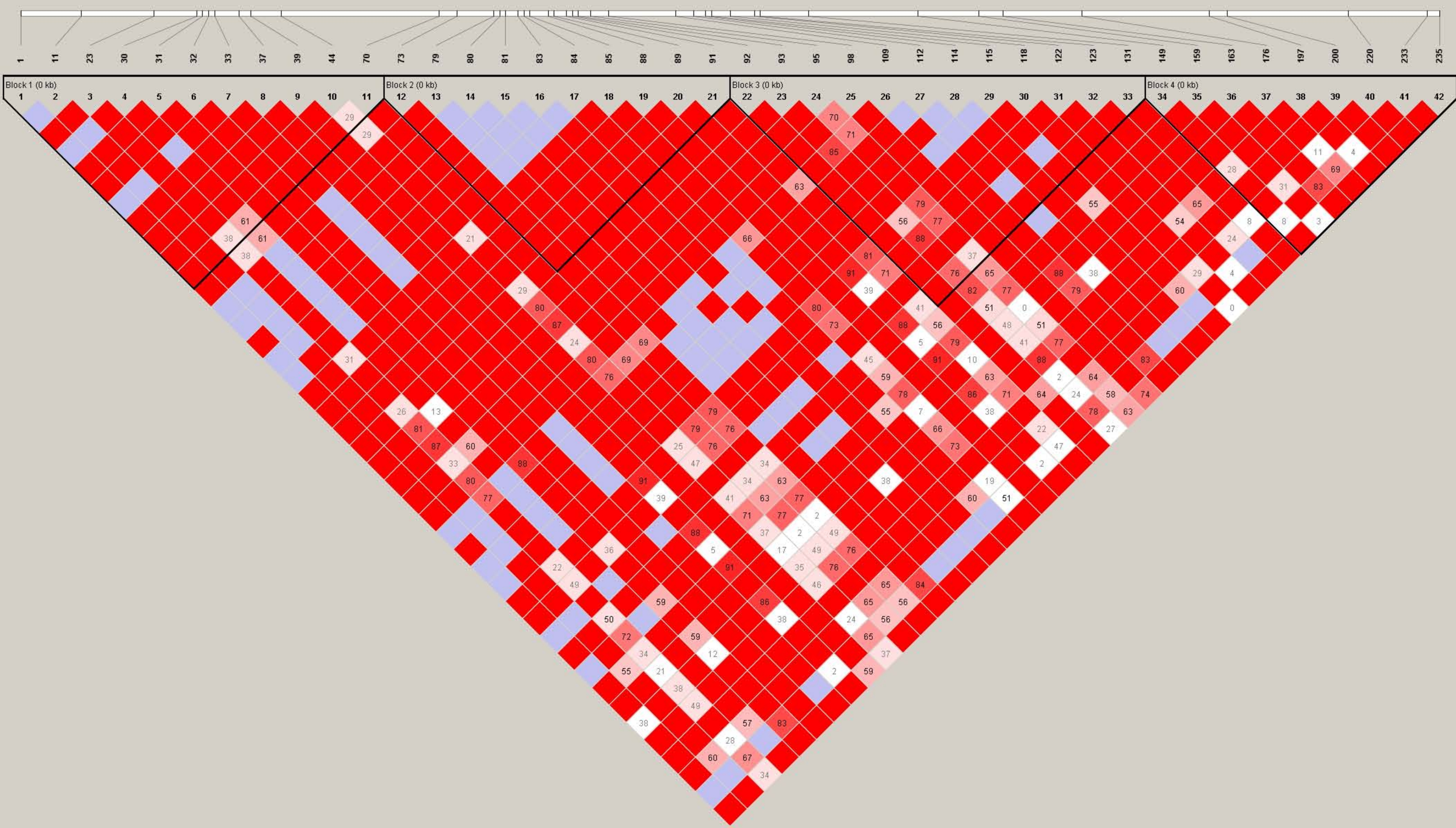

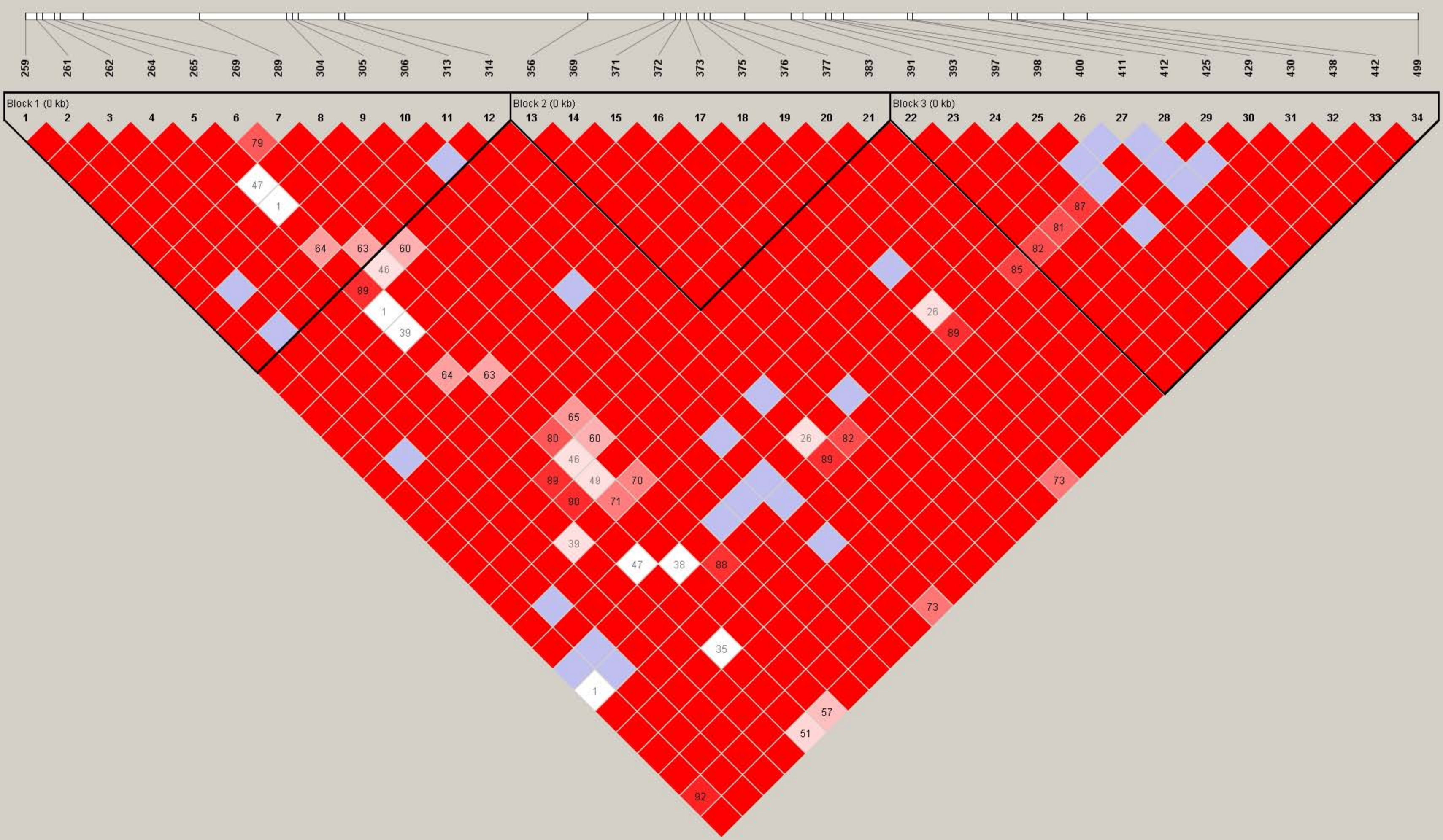

Supplement: Additional file 2 — The complete plot of LD for all tag SNPs. [file 1471-2156-12-48-S2.PDF]
