# Supplementary material for: Combining an Evolution-guided Clustering Algorithm and Haplotype-based LRT in Family Association Studies
Source: BMC Genet. 2011 May 19;12:48. doi: 10.1186/1471-2156-12-48 (PMC3118131; doi:10.1186/1471-2156-12-48)
Supplement: Additional file 3 — Derivation of P(A|D). [file 1471-2156-12-48-S3.PDF]

Additional file 2

Derivations of $P(A|D)$

Let the allele frequencies be $P(A)=p$ and $P(a)=1-p$, the genotype frequencies be $P(AA)=p^2$, $P(Aa)=2p(1-p)$, and $P(aa)=(1-p)^2$, the penetrance $f_0=P(D|aa)$, $f_1=P(D|Aa)$ and $f_2=P(D|AA)$, and the prevalence $K=P(D)$, then the allele frequency conditioning on diseased individuals $P(A|D)$ is

$$
\begin{aligned}
P(A|D) &= P(A\cap AA|D)+P(A\cap Aa|D)+P(A\cap aa|D) \\
&= P(A|AA\cap D)P(AA|D)+P(A|Aa\cap D)P(Aa|D)+0 \\
&= P(AA|D)+P(A|Aa)P(Aa|D)+0 \\
&= P(AA|D)+\frac{1}{2}P(Aa|D)
\end{aligned}
$$

Now apply Bayes' theorem to both conditional probabilities above,

$$P(AA|D)=P(D|AA)P(AA)/P(D)=f_2\cdot p^2/K$$

$$P(Aa|D)=P(D|Aa)P(Aa)/P(D)=f_1\cdot 2p(1-p)/K$$

Therefore, the conditional allele frequency becomes

$$P(A|D)=f_2\cdot p^2/K+\frac{1}{2}f_1\cdot 2p(1-p)/K\ .$$
